# Military Personals

**Published:** 1917-12

**Authors:** 


### MILITARY PERSONALS

Dr. R. C. Miller of Buffalo has been appointed Assistant Surgeon U. S. Naval Reserve and has been assigned to duty in the Recruiting Station in Buffalo.

Dr. Regina Flood-Keyes of Buffalo left about November first for Paris, her ultimate destination being Servia where she will serve as Surgeon in the American Red Cross.

Dr. Clarence H. Mackay of Lancaster returned to his studies in Boston Oct. 27, after a week's leave at home.

Dr. Leo C. Reimann of Franklinville went to Washington about Nov. 1, for a course in military medicine, preparatory to service abroad.

Lt. J. Arthur Elson of Albion has been ordered to duty at Ft. Riley, Kas.

Lt. Ralph E. Brodie of Albion who has been at the Yaphank training camp, expects to be ordered to France soon.

Major John W. Meehan of the regular Medical Corps, who has been stationed at Syracuse, was ordered to Ft. Sheridan, Ill., Nov. 1, to organize a hospital train.

Lieut. Charles W. Strowger of Pittsford writes from France Oct. 8. He tells of traveling on the military tickets which are one quarter the regular fare, (about ½ cent a mile) officers traveling first class without extra expense.

Major Charles W. Hennington of Rochester, our associate editor, wrote last month from Boston that he was in a class of twenty, studying military orthopaedics with Lovett, Mark Rogers and others. The course is directed largely by the experience of Robert Jones of Liverpool with British Troops.

Capt. Charles H. Erway of Elmira has been ordered to Camp Hancock, Augusta, Ga., for the special examination of the command for tuberculosis.

Lt. Francis A. Georger of Buffalo arrived in France in November.

Capt. Kent E. Williams of Rome has been ordered to Camp Funston, Fort Riley, Kansas.

Lieuts. Milton G. Burch, Hornell, Clinton E. Goodwin, Weedsport, Lewell T. Genung, Worchester, George H. Shaw, Camillus, have been ordered to Camp Sherman, Chillicothe, Ohio.

Lieut. Arthur B. Graves, Perrysburg, has been ordered to Camp Wheeler, Macon, Ga.

Lieut. Daniel E. L. Stedem, Buffalo, has been ordered to Fort Oglethorpe for instruction.

To Army School, Lieut. Leo E. Reimann, Buffalo.

To Camp Funston, Fort Riley, Kan., Major Martin B. Tinker, Ithaca.

To Camp Grant, Rockford, Ill., Lieuts. Minor McDaniels, Ithaca; Allen N. Moore, Lockport; Otto J. von Renner, Buffalo; John L. Bishop, Niagara Falls.

To Camp Lee, Petersburg, Va., Lieut. Forest R. Mildrew, Auburn.

To Camp McClellan, Annistan, Ala., Lieut. Frederick C. Devendorf, Utica.

To Camp Meade, Annapolis Junction, Md., Lieut. John R. Booth, Rochester.

To Camp Devens, Ayer, Mass., Lieuts. Francis J. Talbot, Niagara Falls; Robert Knight, Seneca Falls; Benjamin F. Colegrove, Van Etten; James B. Foster, Webster.

To Fort Niagara, Major Frederick N. C. Jerauld, Niagara Falls.

To Boston City Hospital, Lieuts. Arthur H. Nylen, Brooklyn; Edmund B. Spaeth, Elmira.

To Camp Custer, Battle Creek, Mich., Lieut. Roswell F. Foster, Westfield.

To Camp Greenleaf, Fort Oglethorp, Lieuts. Robert P. Dobbie, Buffalo; Wm. W. Carleston, Waterloo.

To Camp Shelby, Hattiesburg, Miss., Capt. Clarence E. Coon, Syracuse.

To Fort Benjamin Harrison, Ind., Lieut. Adelbert C. Abbott, Syracuse.

To Fort Leavenworth, Kan., Lieut. Noah P. Norman, Watkins.

To Mineola, L. I., Aviation Section, Lieut. Scott R. Fisher, Syracuse.

To Syracuse Re-Organization Camp, Lieut. Howard C. Fairbanks, Tonawanda.

Camp Sheridan, Montgomery, Ala., Capt. A. L. Benedict, Buffalo, Gastro-enterologist, 37th Division.

To Kelly Field, Signal Corps, Aviaton School, SanAntonio, Texas, Lieuts. Joseph Brumberg and Milton H. Goldberg, Buffalo.

Camp Custer, Battle Creek, Mich., Capt. George F. Mills, Oneida.

Camp Devens, Ayer, Mass., Lieuts. Charles E. Congdon, Buffalo; Adelbert C. Abbott, Syracuse.

Camp Greenleaf, Fort Oglethorpe, Lieut. Warren C. Fargo, Warsaw.

Camp Joseph E. Johnston, Jacksonville, Fla., Lieut. Macy L. Lerner, Rochester.

Camp Pike, Little Rock, Ark., Lieut. Robert R. McCully, Auburn.

Camp Upton, Yaphank, L. I. N. Y., Lieut. Albert C. Durand, Ithaca.

Chicago, Ill., Neurological School, Major Charles Hennington.

Fort Oglethorpe, Lieut. Harold B. Johnson, Buffalo.

Fort Sill, Okla., Lieut. Edwin S. Ingersoll, Rochester.

Langley Field, Hampton, Va., Edward L. Hezeltune, Jamestown.

New York City, Bellevue Hospital, Capt. Chas. W. Hoyt, Rochester; Lieut. Bernard S. Strait, Penn Yan.

Walter Reed General Hospital, Takoma Park, D. C., Capts. Joseph R. Culkin, Rochester; Stephen A. Mahady, Utica.

Honorably discharged on account of physical disqualification, Lieut. John W. Munro, Rochester.

Lieut. A. E. McCarthy has been transferred from Camp Benj. Harrison to Camp Taylor, Louisville, Ky. Special work Tuberculosis.

